# The expression of CXCR4/CXCL12 determines subsets of patients in systemic lupus erythematosus

**DOI:** 10.1186/1479-5876-9-S2-P60

**Published:** 2011-11-23

**Authors:** Philippe Guilpain, Andrew Wang, Benjamin F Chong, Sandrine Chouzenoux, Loic Guillevin, Xin J Zhou, Fangming Lin, Anna-Marie Fairhurst, Christopher Boudreaux, Christian Roux, Edward K Wakeland, Laurie Davis, Frederic Batteux, Chandra Mohan

**Affiliations:** 1 Médecine Interne A, Hôpital Saint-Eloi, Université Montpellier 1, Montpellier, France; 2 Laboratoire d'immunologie, EA 1833, Université Paris 5, Cochin, Paris, France; 3 Médecine Interne, Hôpital Cochin, Université Paris 5, Paris, France; 4 University of Texas Southwestern Medical Center, Dallas, TX, USA; 5 Rheumatology, Hôpital Cochin, Université Paris 5, Paris, France

## Background

CXCR4 is a chemokine with numerous effects on the immune system. In murine lupus models, we observed an overexpression of CXCR4, and its ligand, CXCL12, in patients with kidney disease. We assessed whether CXCR4 and CXCL12 were enhanced in human systemic lupus erythematosus (SLE) peripheral blood leukocytes (PBLs) and kidneys.

## Methods

PBLs from 24 consecutive SLE patients were prospectively analyzed by flow cytometry for CXCR4 expression. Human lupus nephritis biopsies (n=16) were immunostained with anti-CXCL12 antibody.

## Results

B cells and CD4^+^ T cells from SLE patients exhibited an over two-fold increase (p<0.005) and three-fold increase (p<0.0005), respectively, in median CXCR4 expression compared to controls (n=8). Moreover, CXCR4 expression on B cells was positively associated with disease activity and increased by 1.61-fold in patients with Systemic Lupus Erythematosus Disease Activity Index (SLEDAI) scores >10 compared to those with SLEDAI scores ≤10 (p=0.005), and was 1.36-fold higher in active neuropsychiatric SLE (NPSLE) patients compared to non-NPSLE patients (p=0.11). CXCL12 was significantly up-regulated in lupus nephritis kidneys (n=16), with the extent and level of expression was correlated with histopathological scores.

## Conclusions

CXCR4 is up-regulated in multiple leukocyte subsets in SLE patients, especially in those with increased disease severity. The heightened expression of CXCR4 in B cells in NPSLE, and CXCL12 in nephritic kidneys suggest that the CXCR4/CXCL12 axis might be a potential target for therapeutic intervention for patients with renal SLE and/or NPSLE.

